# Effects of Different Anti-Epileptic Drug Groups and Brushing on the Color Stability of Restorative Materials Used in Pedodontics: An In Vitro Evaluation

**DOI:** 10.3390/children11020235

**Published:** 2024-02-11

**Authors:** Mehmet Sinan Dogan, Şemsettin Yıldız

**Affiliations:** 1 Department of Pediatric Dentistry, Faculty of Dentistry, Harran University, Sanliurfa 63050, Turkey; 2 Department of Pediatric Dentistry, Faculty of Dentistry, Fırat University, Elazıg 23119, Turkey; semsettin.yildiz@firat.edu.tr

**Keywords:** anti-epileptic drugs, dental materials, tooth brushing, color stability spectrophotometer

## Abstract

Objectives: This study will evaluate the effects of anti-epileptic drugs and brushing used in children on the color change of three restorative materials by creating an in vitro study model. Methods: Forty samples of polyacid-modified composite resin (compomer), glass ionomer cement (GIC), and composite resin (CR) were prepared. Samples were split into four groups (n = 10) and soaked in three anti-epileptic drugs (Tegretol, Depakine, Keppra) and distilled water. For each group (n = 5), two subgroups (brushing and non-brushing) were created. Discolorations [CIEDE2000 (ΔE_00_)] were determined initially and on days 7 and 14. The data were analyzed with a four-factor repeated measures ANOVA analysis, and a post hoc analysis Bonferroni test was used. Results: After the second week, the highest ΔE_00_ value was seen in the non-brushed compomer material in the Tegretol drug group (8.59 ± 0.43). In contrast, the lowest value was seen in GIC filling material-brushing-Depakine drug (3.45 ± 2.14). ΔE_00_ values in the brushing groups were statistically significantly lower than those in the no brushing groups (*p* < 0.05). Conclusions: It has been determined that the color stability of aesthetic restorative dental materials used in pediatric dentistry is affected by antiepileptic drugs. In addition, it has been determined that tooth brushing positively affects the color stability of restorative materials. Therefore, pediatric dentists should advise their patients and their relatives about this issue and take precautions.

## 1. Introduction

Having an aesthetic appearance has an increasingly important place in today’s dentistry practices in line with the demands of patients [1]. With increasing awareness, the aesthetic expectations of parents and children also increase. For this reason, dentists are often consulted for aesthetic reasons for the unpleasant appearance of teeth due to decay and trauma [2]. Giving the patient an aesthetic appearance can be considered a primary factor for social acceptance and professional success [3].

The treatment of decayed primary teeth has a significant impact on child health. Due to aesthetic concerns, the use of tooth-colored restorative materials, such as CR, GIC, and compomers, has increased in pediatric dentistry [4]. Thanks to their superior properties, these three materials are widely used in both anterior and posterior teeth [5]. The restorative materials’ color stability is an important criterion in the long-term success of the selected restorations [6]. However, discoloration may occur in the restorations over time due to intrinsic and extrinsic factors. In intrinsic staining, the resin matrix content may be related to particle proportion and size. In extrinsic coloration, polymerization shrinkage can be attributed to food and beverage consumption and drug formulations containing colorants/additives [7]. 

Liquid oral medications are generally prescribed for the long-term treatment of chronic diseases or acute situations in children [8]. Medications contain components such as sugars, acids, buffering agents, and water- and oil-soluble coloring agents in these liquid forms [9,10]. Epilepsy, a chronic disease, is a common neurological disease seen in children. Symptoms often begin in childhood [11]. Children with epilepsy must constantly use various prescribed syrups and oral suspensions [12]. 

Acidic pediatric drug liquids cause a decrease in enamel hardness in primary teeth and cause surface disintegration and surface changes of restorations [13].

The long-term clinical success of restorative materials (such as GIC, compomer, and CR) depends on the absence of discoloration. They are negatively affected by factors such as the frequent consumption of colored foods and beverages and long-term drug use. Thus, not fully meeting the aesthetic expectations of the patient and the parent makes the clinician’s job difficult [12]. Pediatric drugs, especially in syrup form, can change the color of dental restorations [14,15]. Some studies have stated that antiepileptic drugs (Sodium Valproate and Tegrotol) have negatively affected dental fillings [12,14,15]. Different studies have been conducted on whether pediatric medications frequently used in children affect the color of aesthetic fillings [16,17]. However, no study has assessed the effect of only antiepileptic drug groups and brushing on discoloration of restorative materials. Thus, this study aims to determine the effect of antiepileptic drugs on the color stability of GIC, compomer, and CR by creating an in vitro study model. It also aims to investigate the effectiveness of brushing on samples with discoloration.

Three null hypotheses were considered: H0, tooth brushing will not affect the discoloration of GIC, compomer, and CR; H1, the type of material does not affect color change; and H2, exposure and duration of exposure to other anti-epileptic pediatric drugs do not affect the color change of GIC, compomer, and CR.

## 2. Material and Methods

In the study, we used three dental restorative materials and three antiepileptic liquid drug groups that are frequently prescribed to children. They are summarized in Table 1 and Table 2. A2 color of the restorative materials was chosen for standardization purposes.

### 2.1. Preparation of Samples

Using ring molds, 40 disk-shaped samples (10 mm diameter × 2 mm thickness) were obtained from each material. A transparent matrix tape was placed over the ring and the samples were placed between two 1 mm thick glass plates to prevent air trapping and voids.

One hundred and twenty restorative material samples were prepared and placed in accordance with the manufacturer’s instructions.

Polymerization of CR and compomer samples was carried out on the upper and lower surfaces with a light-emitting diode device for 20 s (Guilin Woodpecker Medical Instrument Co, Guilin, China). In this process, the light device was made by contacting the glass surfaces.

For rapid-setting conventional glass ionomer cement (GIC), each sample was prepared by mixing in an amalgam mixer (Gnatus Amalgam mix 2) for 10 s (according to the manufacturer’s instructions). The sample materials were placed in the disk with the help of a carrier. It was allowed to polymerize chemically. Restorations were removed from the disks. All samples with irregularities or voids in their shape or surface were excluded from evaluation.

**Figure 1:**
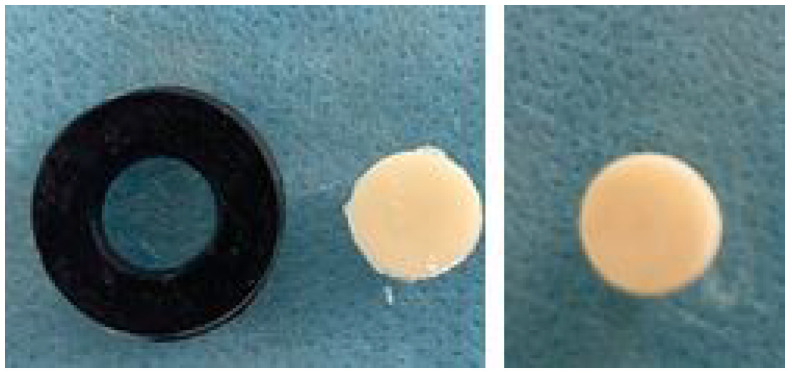
Disk-shaped finished and polished samples.

Aluminum oxide discs were used for polishing and finishing all samples (coarse, medium, fine, and super-fine-grain Sof-Lex Polishing Discs, 3M ESPE, St. Paul, MN, USA). A separate polishing disk was used for each sample (Figure 1). After polishing, each material was rinsed thoroughly under running distilled water for 10 s to remove debris. As conducted in the previous study, all samples were kept in distilled water at 37 °C for 24 h. In this way, the polymerization processes were completed [16].

### 2.2. Subgrouping of the Specimens

Polishing and finishing processes were completed before basic color measurements were taken. Samples were randomly separated into four groups (n = 10). Distilled water (pH 6.47) was used as the control solution. For each group (n = 5), two subgroups (brushing and not brushing) were created (Table 3 and Table 4). Considering a previous study [12], according to the results of power analysis performed with the G * Power program (G * Power 3.1 software; Heinrich Heine University, Düsseldorf, Germany; F test, ANOVA: for fixed effects, special, main effects and interactions analyses), 3 × 4 = 12 groups were divided into two subgroups: brushing and not brushing. The 24 groups were formed with α (margin of error) = 0.05 and 0.45. It was determined that a minimum of 120 and 5 samples in each subgroup were sufficient at the effect (f) 0.95 power (1-β) level.

### 2.3. Discoloration Measurement and Brushing Cycles

**Figure 2:**
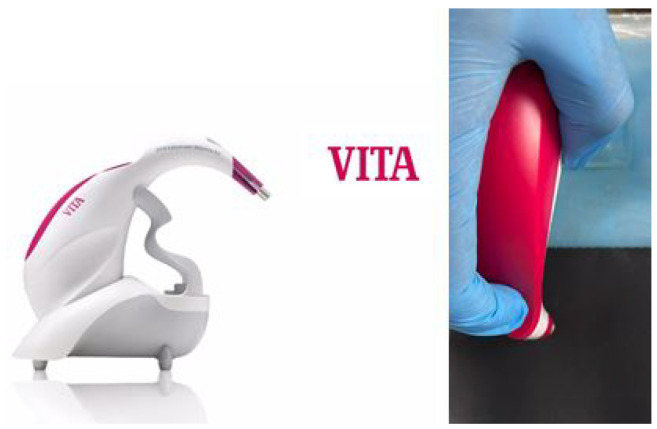
In the study, a spectrophotometer was used.

The first color measurement after polymerization of the samples was made under the same terms with the help of a contact spectrophotometer (VitaEasyshade^®^V, VITA Zahnfabrik GmbH&Co. KG, Bad Säckingen, Germany) (Figure 2). After the first color measurements, the samples kept in distilled water were exposed to 3 drug groups (10 mL undiluted) for 1 min, 3 times a day (8 h apart). This process was applied for two weeks, and the solutions were renewed after each immersion. Between immersion periods, the samples were stored in distilled water at room temperature. The temperatures of all solutions were controlled using a thermometer (Versatile, VCD-800, Beijing, China).

Color measurements of the samples were made according to the CIEDE2000 scale. Each sample was measured three consecutive times with a spectrophotometer calibrated as recommended by the manufacturer. The averages of the measurements were included in the analysis. Total color difference (ΔE_00_) was determined using the equation below:

$$\Delta E_{00}=\;\sqrt{{\left(\frac{\Delta L\prime }{kLSL}\right)}^{2}+{\left(\frac{\Delta C\prime }{kCSC}\right)}^{2}+{\left(\frac{\Delta H\prime }{kHSH}\right)}^{2}+RT\frac{\Delta C\prime }{kCSC}\frac{\Delta H\prime }{kHSH}}$$

Samples in the brushing group were brushed daily using fluoride-free toothpaste and an electric toothbrush (R.O.C.S Kids, Banana Mix, Tallinn, Estonia- Oral B Genius X White Braun, Kronberg, Germany). To simulate tooth brushing at home, applying 2 mL of toothpaste to each sample surface, both surfaces were brushed using the same electric toothbrush in “daily cleaning” mode, using 40 strokes with a force of 2 N. After scrubbing, the sample was washed with distilled water and placed in distilled water until further processing. Color measurements were made after the samples were dried with paper tissues. Colorations were measured at baseline and after 7 days and 14 days.

### 2.4. Analysis of Data

Within the scope of the study, the SPSS 23.0 (SPPS Inc., Chicago, IL, USA) package program was used for statistical analyses. In the study, values are stated as mean ± standard deviation. Data were examined by four-factor repeated measures ANOVA (analysis of variance) analysis using the general linear model procedure for repeated measures. The model included “material”, “solution”, “scrubbing condition”, and “time” and their interaction terms as main effects. A post hoc analysis Bonferroni test was used. Statistical importance was accepted as *p* < 0.05 in the entire study.

## 3. Results

**Figure 3:**
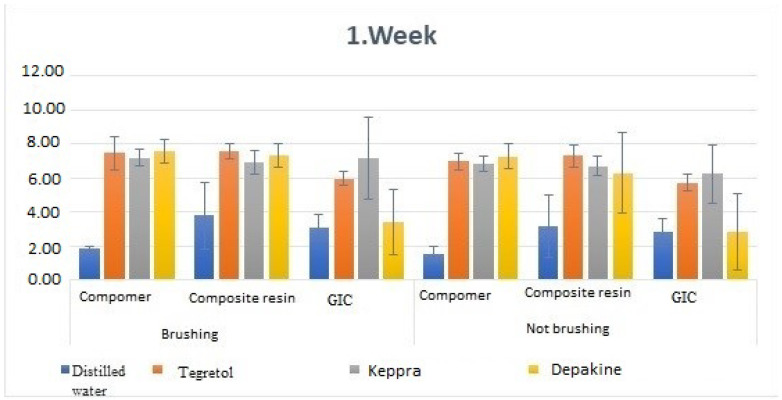
Comparison of week 1 discoloration value measurements.

To reveal the effects of the material, solution, and brushing conditions on the change in discoloration values over time, the data were examined with 3(Material) × 4(Solution) × 2(Brushing Condition) and one repeated measures ANOVA. According to the results, time statistically affects the discoloration value (F: 103.101, *p*: 0.001). Similarly, the type of solution also has a statistically significant impact on the discoloration value (F: 49.735, *p*: 0.001). The brushing condition statistically affects discoloration values (F: 9.482, *p*: 0.002). It has been revealed by statistical analysis that all four factors have an individual effect. When looking at the impact of interactions, week and solution interaction statistically affect discoloration measurements (F: 30.985, *p*: 0.001). Week and material interaction statistically affect discoloration measurements (F: 15.395, *p*: 0.001). Solution and material interaction statistically affect discoloration measurements (F: 37.782, *p*: 0.001). The triple interaction of week, solution, and material statistically affects discoloration measurements (F: 9.510, *p*: 0.001). The total interaction of four factors has a statistically important effect on discoloration measurements (F: 0.100, *p*: 0.996). The explanation rate of the established model for the discoloration value is 73.2% (Adjusted R2 = 0.732) (Table 3, Figure 3).

**Figure 4:**
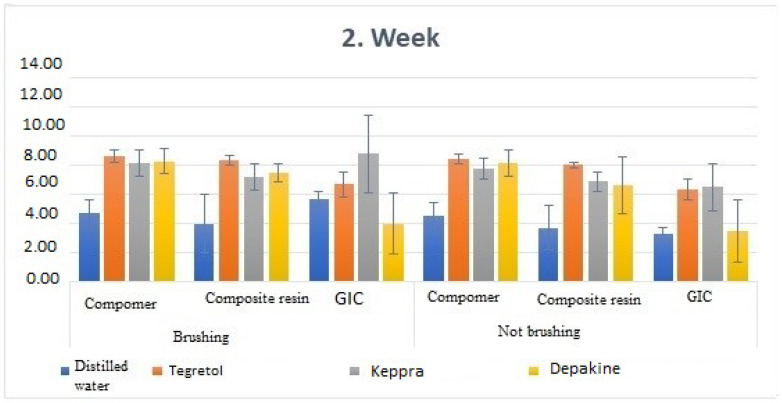
Comparison of week 2 discoloration value measurements.

When comparing the brushed and non-brushed samples with compomer material according to the solutions in the first-week measurement, a statistically important difference was found in both cases (*p* < 0.05). It was determined that the lowest measurement value was in distilled water, and the highest measurement value was in Depakine (Sodium Valproate) solution (Table 4, Figure 3 and Figure 4).

When comparing the brushed and unbrushed samples with composite material according to the solutions in the first week of measurement, a statistically important difference was found in both cases (*p* < 0.05). It was determined that the lowest was in distilled water and the highest was in Tegretol (Carbamazepine) solution.

A statistically important difference was found in both cases when comparing the brushed and unbrushed samples with GIC material according to the solutions in the first week of measurement (*p* < 0.05). It was found that the lowest measurement value was in Depakine (Sodium Valproate) solution in the non-brushing group, while it was in distilled water in the brushing group, and the highest measurement value was in Keppra (Levetiracetam) solution in the brushed/non-brushing group.

When comparing the brushed and unbrushed samples with compomer and composite materials according to the solutions in the second-week measurement, a statistically important difference was found in both cases (*p* < 0.05). It was determined that the lowest was in distilled water and the highest was in Tegretol (Carbamazepine) solution (Table 4).

When comparing the unbrushed samples with GIC material to the solutions in the second week of measurement, a statistically important difference was found (*p* < 0.05). It was determined that the lowest measurement value was in Depakine (Sodium Valproate), and the highest measurement value was in Keppra (Levetiracetam).

A statistically important difference was found between the first and second-week measurements of the samples kept in distilled water, Tegretol (Carbamazepine), and Keppra (Levetiracetam) among the unbrushed samples with compomer material (*p* < 0.05), but there was no statistically important difference between the first and second-week measurements of the samples kept in Depakine (Sodium Valproate) (*p* > 0.05). Among the brushed samples containing compomer material, a statistically important difference was found between the first and second-week measurements of the samples kept in distilled water, Tegretol (Carbamazepine), Keppra (Levetiracetam), and Depakine (Sodium Valproate) (*p* < 0.05).

While there was no statistically important difference between the first and second-week measurements of the samples kept in distilled water, Tegretol (Carbamazepine), Keppra (Levetiracetam), or Depakine (Sodium Valproate) among the unbrushed samples with composite material (*p* > 0.05), a statistically important difference was determined in the samples kept in Tegretol (Carbamazepine) (*p* < 0.05).

Among the unbrushed samples with GIC material, a statistically important difference was found between the first and second-week measurements of the samples kept in distilled water, Tegretol (Carbamazepine), and Keppra (Levetiracetam) (*p* < 0.05), while there was no statistically important difference for the Depakine (Sodium Valproate) group (*p* > 0.052). There was a statistically important difference between the first and second-week measurements of the brushed samples with GIC material and kept in distilled water, Tegretol (Carbamazepine), and Depakine (Sodium Valproate) (*p* < 0.05). No statistically important difference was found between the first and second-week measurements of the samples kept in Keppra (Levetiracetam) among the brushed samples with compomer material (*p* > 0.05).

A statistically important difference was found when comparing the brushed and non-brushed samples kept in Tegretol (Carbamazepine) solution according to the materials in the first-week measurement (*p* < 0.05). It was found that the lowest measurement value was in GIC, and the highest was in composite.

No statistically important difference was determined when comparing the brushed and unbrushed samples kept in Keppra (Levetiracetam) solution according to the materials in the first-week measurement (*p* > 0.05).

A statistically important difference was found when comparing the brushed and non-brushed samples kept in Depakine (Sodium Valproate) and Tegretol (Carbamazepine) solutions according to the materials in the first week of measurement (*p* < 0.05). It was found that the lowest measured value was in GIC, and the highest calculated value was in compomer.

A statistically important difference was found when comparing the unbrushed samples kept in Keppra (Levetiracetam) solution according to the materials in the second-week measurement (*p* < 0.05). It was determined that the lowest measurement value was in CR, and the highest measurement value was in GIC.

In the second week of measurement, a statistically important difference was found when the brushed samples kept in Keppra (Levetiracetam) and Depakine (Sodium Valproate) solutions were compared according to the materials (*p* < 0.05). It was found that the lowest measurement value was for GIC, and the highest measurement value was for compomer.

When comparing the discoloration difference between the groups in the second week to the first week, it was determined that the highest discoloration value occurred in the brushed and non-brushed compomer material samples kept in distilled water.

When comparing the discoloration difference between the groups in the second week to the first week, it was determined that the lowest discoloration value occurred in the samples of the non-brushed composite material kept in Depakine (Sodium Valproate) (Table 5).

## 4. Discussion

In this study, the effect of tooth brushing on the discoloration of CR, GIC, and compomer was evaluated after one week and two weeks of exposure to common pediatric anti-epileptic drugs. The brushing condition was determined as having a statistically significant effect on discoloration values, and the H0 hypothesis was rejected. The H1 hypothesis was also rejected because there were differences in discolorations among the restorative materials used in the study compared with the pediatric drugs tested. Additionally, the discoloration over time differed for each drug investigated, thus rejecting the H2 hypothesis.

The CIELAB color determination system was most frequently used in dentistry. The International Commission on Illumination (CIE) has recommended using CIEDE2000 (Δ*E*_00_), a new color difference formula invented by Munsell, since 2001 [18]. This formula has been used to determine discoloration since 2013. It has been accepted as a standard formula [19]. Digital color measurement devices were used to observe discolorations in restorative materials [20]. Studies have also reported that Vita Easyshade is the most frequently used device among many spectrophotometers because it is repeatable and reliable in color determination [21,22,23,24]. Therefore, in our study, to evaluate the discoloration of restorative materials, the Δ*E*_00_ formula was used, and Vita Easyshade Advance 4.0 was used for color define.

Paravina et al. reported that color differences were compared with 50:50% detectability (PT) and 50:50% acceptability (AT) thresholds, and PT (0.81 units) and AT (1) for CIEDE2000 (1:1:1) (0.80 units) values were obtained. In addition, Δ*E*_00_ < 1.8 indicates that it is a clinically acceptable change [25]. In this study, while discoloration was found in the compomer filling material in the distilled water group brushed in the first week, it was observed that it did not give acceptable values in the other groups and restorative materials in the first and second weeks.

An important factor affecting the aesthetic properties of dental restorative materials used in children is the absence of long-term color change. Dental treatments that require repeated visits to the dentist and the replacement of restorations due to aesthetic concerns are costly for the patient and may increase the child’s dental anxiety [12,26,27]. In the literature, studies have been conducted investigating the effects of medications frequently used by children and brushing on the discoloration of restorative materials [3,12,16,17,24,25,26,27,28,29,30,31]. No study evaluates only anti-epileptic drug groups.

To simulate the study accurately, as reported in similar studies, samples were placed in anti-epileptic drugs for 1 min every 8 h and then similarly stored in distilled water [16,17]. One study stated that the artificial saliva storage environment is not a clinically appropriate environment. Additionally, resin-based materials stored in artificial saliva or distilled water were similar [32]. For this reason, in our study, we used distilled water instead of artificial saliva to store the samples.

Studies have shown that GICs are the most resistant restorations to coloration due to their high water content [33,34]. However, it is thought that discolorations in resin-containing materials can be relevant to chemical changes in the initiator system, water absorption in the composite monomer, and the activator [30,33,35].

One reason for discoloration in restorative dental materials is water absorption [36,37]. Dental materials that absorb water can easily absorb other liquids that cause discoloration [33,38]. It has been stated in a previous study that GIC’s absorb less water due to their high water content [33].

Tüzüner et al. found that the greatest discoloration occurred in CR after exposure to pediatric drugs. In contrast, the smallest change was observed in the GIC material. GIC has been found to have acceptable color stability across pediatric drug groups evaluated compared to composite or compomer materials [30].

In a study conducted by Srvastava et al. on the color change of antiepileptic drugs, they found that the greatest color change was in resin-reinforced glass ionomer (between zirconia-reinforced glass ionomer, composite, and resin-reinforced glass ionomer) [12]. This research provides results that support our study. Our study observed the lowest discoloration in the second week in the brushed GIC material kept in Depakine (Sodium Valproate). In addition, it has been observed that resin-containing composite and compomer restorations preserved in Keppra (Levetiracetam) have less discoloration than Depakine (Sodium Valproate).

Jamel et al., in a study related to the color stability of various pediatric drugs (multivitamins, bronchodilator, antiepileptic, antibiotic, and distilled water) and three resin-containing restorative materials (composite resin, compomer, and resin-reinforced GIC), determined that the drugs affect the color stability of the restorations. In addition, it has been observed that the color stability of restorations is mostly negatively affected by bronchodilators and antiepileptic drugs, and the color stability of compomer is better than other restorations [14].

Tüzüner et al., Bezgin et al., Bagheri et al., Tunc et al., Nalwade et al., and Faghihi et al. stated that GIC material has better color stability against drugs than resin-containing materials [5,28,30,33,34,39]. The results of our study are parallel to these studies. Additionally, it was observed that the lowest values in GIC (except the Depakine group), compomer, and composite groups were in the distilled water group. In general, it has been observed that drugs affect the color stability of restorations.

Contrary to these studies, there are studies stating that GIC (hydrophilic materials) is more prone to coloration than resin-containing composites (hydrophobic materials) [40,41,42]. Almutairi et al. observed that GIC showed more discoloration than composites and compomers. Adusumilli et al. also obtained similar results [3,16]. In the literature review, there are two opinions regarding the differences in the discolorations of restorative dental materials (GIC, compomer, and CR) commonly used in pediatric dentistry and pediatric drugs. These opinions have been examined above.

The results obtained in this study determined that the lowest discoloration in all groups except Keppra was observed in the GIC group in the first and second weeks. In contrast, the highest discoloration was found in the composite in the Tegretol group in the first week, while the compomer showed the highest discoloration in the Keppra and Depakine groups. In the second week, the highest value was observed in the compomer filling material in the Tegretol drug group. Moreover, at the end of the second week, it was determined that the highest value in GIC material was in the Keppra group. In contrast, the highest value in the composite was observed in the Tegretol group. We think the discoloration of resin-containing restorations in the Tegretol group may be related to the drug’s chemical structure. As a result, throughout the study, the highest color change was found in the compomer, while the lowest value was observed in the GIC material.

A study showed that compomer was more effective against staining caused by pediatric drugs than glass carbomers and glass hydrides. It was observed that Δ*E*00 values were lower in all brushed groups compared to the non-brushed groups. In parallel with these results, in the study on discoloration of restorative materials, it was observed that Δ*E*_00_ was lower in the brushing groups, when pediatric drugs are applied on GIC and compomer [17]. In addition, the surface properties of restorative dental materials are another factor that determines color stability. Fine coloring particles can accumulate in pits on restorative dental materials. Color change, which occurs due to the adsorption of color particles in the pits on the surface, can be prevented by tooth brushing [36,37]. Bezgin et al. concluded that brushing the samples prevented the colorants from adhering to the restorative material and reduced the discoloration over time [39]. The results of our study support this.

Tooth brushing is a critical practice widely used to ensure oral hygiene. Tooth brushing can cause wear on teeth and dental restorations, depending on brushing technique, duration, and force factors. Fluoride particles have negative effects on resin-containing filling materials, and therefore, they recommended using low-abrasive toothpaste that does not contain fluoride for children aged 3–7 years [17]. For this reason, fluoride-free toothpaste and an electric toothbrush with a strength indicator were preferred in this study to standardize the abrasive effect of tooth brushing.

Considering the findings of this study, in vitro studies that mimic oral conditions have shown that restorative dental materials can improve color stability. Taking medications at irregular hours, structural features and components of drugs, and differences in saliva fluid content and buffering capacities of individuals affect the results [33,35,39]. Such in vitro studies provide limited results to researchers as they do not fully mimic the oral environment [30].

## 5. Conclusions

We consider the evaluation of only one brand of each dental restorative material and the generalization of these results to all materials as a limitation of our study. Despite this limitation, we think that the aftereffects of diverse antiepileptic drugs and brushing on the discolorations of CR, compomer, and GIC have not been previously investigated and can be a reference for future studies. Additionally, as a result of our study, it was determined that the discoloration of aesthetic restorative dental materials used in pedodontics was affected by antiepileptic drugs. It was observed that the GIC filling material showed the highest color stability, while the compomer filling material showed the lowest value. In addition, it has been determined that tooth brushing positively affects the color stability of restorative materials. Thus, parents and children should be reminded of the importance of tooth brushing to reduce the adverse effects of antiepileptic drugs on the color stability of materials. It is very important for these patients to visit a pediatric dentist for regular check-ups after restorative treatments.

## Figures and Tables

**Table 1:** In the study, pediatric anti-epileptic drugs were used.

Brand Name	Active Ingredient
Tegretol	Carbamazepine
Depakine	Sodium Valproate
Keppra	Levetiracetam

**Table 2:** Technical properties of the dental materials used in the study.

Product	Material Type	Mixing	Curing	Manufacturer
Dyract	Polyacid- Modified Composite Resin (compomer)	N/A	Light–cure For 20 s	Dentsply DeTrey, GmbH, Konstanz, Germany
XP				
	
Voco	Composite	N/A	Light–cure	Voco GmbH,
Arabesk N	Resin		For	Cuxhaven,
			20 s	Germany
IonoStar	Glass	10 s	No cure, allowed to	Voco,Cuxhaven,
Plus	Ionomer Cement	with a mixer	set for 5 min	Germany

**Table 3:** Four-factor repeated measures ANOVA analysis results.

Source	Type III Sum of Squares	Df	Mean Square	F	*p*	Partial Eta^2^
Corrected Model	1071.748	47	22.803	14.897	0.001 *	0.785
Intercept	9665.466	1	9665.466	6314.283	0.001 *	0.970
Week	157.821	1	157.821	103.101	0.001 *	0.349
Solution	228.395	3	76.132	49.735	0.001 *	0.437
Teeth brushing	14.514	1	14.514	9.482	0.002 *	0.047
Material	35.650	2	17.825	11.645	0.001 *	0.108
Week × Solution	142.289	3	47.430	30.985	0.001 *	0.326
Week × Teeth brushing	0.007	1	0.007	0.004	0.948	0.000
Week × Material	47.131	2	23.565	15.395	0.001 *	0.138
Solution × Teeth brushing	1.602	3	0.534	0.349	0.790	0.005
Solution × Material	347.007	6	57.835	37.782	0.001 *	0.541
Teeth brushing × Material	1.643	2	0.822	0.537	0.586	0.006
Week × Solution × Teeth brushing	1.121	3	0.374	0.244	0.865	0.004
Week × Solution × Material	87.343	6	14.557	9.510	0.001 *	0.229
Week × Teeth brushing × Material	0.752	2	0.376	0.246	0.782	0.003
Çözelti × Teeth brushing × Material	5.553	6	0.925	0.605	0.726	0.019
Week × Solution × Teeth brushing × Material	0.922	6	0.154	0.100	0.996	0.003
Error	293.900	192	1.531			
Total	11,031.114	240				
Corrected Total	1365.648	239				
R^2^ = 0.785 (Adjusted R^2^ = 0.732) * *p* < 0.05						

**Table 4:** Comparison of discoloration value measurements.

		Compomer		Composite		GIC	
		Not-Brushing	Brushing	Not-Brushing	Brushing	Not-Brushing	Brushing
1. Week	Distilled Water	1.85 ± 0.15 ^A,a,1^	1.51 ± 0.44 ^X,a,1^	3.78 ± 1.95 ^A,a,2^	3.14 ± 1.85 ^X,a,2^	3.05 ± 0.76 ^A,a,2^	2.79 ± 0.81 ^X,a,2^
	Tegretol (Carbamazepine)	7.46 ± 0.96 ^B,a,1^	6.97 ± 0.48 ^Y,a,1^	7.58 ± 0.44 ^B,a,1^	7.28 ± 0.65 ^Y,a,1^	5.94 ± 0.41 ^B,a,2^	5.71 ± 0.51 ^Y,a,2^
	Keppra (Levetiracetam)	7.19 ± 0.45 ^B,a,1^	6.84 ± 0.48 ^Y,a,1^	6.92 ± 0.67 ^B,a,1^	6.70 ± 0.59 ^Y,a,1^	7.16 ± 2.38 ^B,a,1^	6.22 ± 1.71 ^Y,a,1^
	Depakine (Sodium Valproate)	7.56 ± 0.73 ^B,a,1^	7.26 ± 0.73 ^Y,a,1^	7.33 ± 0.69 ^B,a,1^	6.29 ± 2.37 ^Y,a,1^	3.41 ± 1.94 ^A,a,2^	2.82 ± 2.22 ^X,a,2^
2. Week	Distilled Water	4.71 ± 0.87 ^A,b,1^	4.53 ± 0.89 ^X,b,1^	3.99 ± 1.97 ^A,a,2^	3.69 ± 1.54 ^X,a,2^	5.65 ± 0.54 ^A,b,3^	3.25 ± 0.48 ^X,b,3^
	Tegretol (Carbamazepine)	8.59 ± 0.43 ^B,b,1^	8.41 ± 0.33 ^Y,b,1^	8.32 ± 0.33 ^B,a,1^	8.04 ± 0.19 ^Y,b,1^	6.68 ± 0.90 ^A,B, b,2^	6.32 ± 0.74 ^Y,b,2^
	Keppra (Levetiracetam)	8.15 ± 0.92 ^B,b,1^	7.78 ± 0.68 ^Y,b,1^	7.16 ± 0.91 ^B,a,2^	6.89 ± 0.66 ^Y,a,2^	8.79 ± 2.65 ^B,b,3^	6.52 ± 1.63 ^Y,a,2^
	Depakine (Sodium Valproate)	8.29 ± 0.83 ^B,a,1^	8.15 ± 0.89 ^Y,b,1^	7.48 ± 0.64 ^B,a,2^	6.63 ± 1.94 ^Y,a,2^	3.98 ± 2.09 ^A,a,3^	3.45 ± 2.14 ^X,a,b,3^

Letters A and B are used to compare the solutions of non-brushed samples of the same material in the same week. Letters X, Y, and Z are the same in the same week. They were used to compare the material samples in the same brushing condition according to the solutions. a and b were used to compare the same solution, same material, and brushing condition over time. Exponential letters 1, 2, and 3 are used to compare samples in the same brushing condition, at the same time, and in the same solution according to materials.

**Table 5:** Comparison of week 2 and week 1 discoloration value measurements.

		Compomer		Composite		GIC	
		Non-Brushing	Brushing	Non-Brushing	Brushing	Non-Brushing	Brushing
2. Week—1. Week	Distilled Water	2.86 ± 0.88	3.02 ± 0.55	0.21 ± 0.14	0.55 ± 0.59	2.6 ± 1.11	0.46 ± 0.24
	Tegretol (Carbamazepine)	1.13 ± 0.88	1.43 ± 0.79	0.75 ± 0.31	0.75 ± 0.83	0.74 ± 0.79	0.60 ± 0.47
	Keppra (Levetiracetam)	0.95 ± 0.73	0.94 ± 0.48	0.24 ± 0.40	0.19 ± 0.13	1.63 ± 0.72	0.29 ± 0.17
	Depakine (Sodium Valproate)	0.73 ± 0.13	0.89 ± 0.31	0.15 ± 0.15	0.34 ± 0.46	0.57 ± 0.45	0.63 ± 0.57

## Data Availability

The data presented in this study are available on request from the corresponding author. The data are not publicly available due to privacy.

## References

[1] Aiem E., Smail-Faugeron V., Muller-Bolla M. (2017). Aesthetic preformed pediatric crowns: Systematic review. Int. J. Paediatr. Dent..

[2] Tarang C., Gunjan Y., Mani T.A., Kavita D., Arora D. (2017). Recent trends of esthetics in pediatric dentistry. Int. J. Oral Health Med. Res..

[3] Adusumilli H., Avula J.S., Kakarla P., Bandi S., Mallela G.M., Vallabhaneni K. (2016). Colour stability of esthetic restorative materials used in pediatric dentistry: An in vitro study. J. Indian Soc. Pedod. Prev. Dent..

[4] Krämer N., Lohbauer U., Frankenberger R. (2007). Restorative materials in the primary dentition of poli-caries patients. Eur. Arch. Paediatr. Dent..

[5] Kale Y.J., Nalwade A.V., Dahake P.T., Dadpe M.V., Kendre S.B. (2019). Effect of different pediatric drug formulations on color stability of composite, zirconia-reinforced glass ionomer cement, and glass ionomer cement. J. Indian Soc. Pedod. Prev. Dent..

[6] Shamszadeh S., Sheikh-Al-Eslamian S.M., Hasani E., Abrandabadi A.N., Panahandeh N. (2016). Color stability of the bulk-fill composite resins with different thickness in response to coffee/water immersion. Int. J. Dent..

[7] Llena C., Fernandez S., Forner L. (2017). Color stability of nanohybidresin-based composites, ormocers and compomers. Clin. Oral Investig..

[8] Khatri A., Nandlal B. (2010). Staining of a conventional and a nanofilled composite resin exposed in vitro to liquid ingested by children. Int. J. Clin. Pediatr. Dent..

[9] Headley J., Northstone K. (2007). Medication administered to children from 0 to 7.5 years in the Avon longitudinal study of parents and children (ALSPAC). Eur. J. Clin. Pharmacol..

[10] Cavalcanti A.L., De Sousa R.I., Clementino M.A., Vieira F.F., Cavalcanti C.L., Xavier A.F. (2012). In vitro analysis of the cariogenic and erosive potential of pediatric antitussive liquid oral medications. Tanzan. J. Health Res..

[11] Guerrini R. (2006). Epilepsy in children. Lancet.

[12] Srivastava V., Jawdekar A. (2022). Comparative Evaluation of Colour Changes In Composite Resin Restorative Material, Resin Reinforced Glass Ionomer Restorative Material and Zirconia Reinforced Glass Ionomer Restorative Material Caused by Three Pediatric Liquid Formulations Prescribed in Epileptic Disorders: An in-Vitro Study. NeuroQuantology.

[13] Yoonis E., Kukletová M. (2009). Tooth-colored dental restorative materials in the primary dentition. Scr. Med..

[14] Jamal D.Y., Farsi N.M., El-Housseiny A.A., Felemban O.M. (2022). Effects of pediatric liquid medications on surface properties of dental restorations. Med. Sci..

[15] Singh A., Grover C., Raina D., Pandey A., Sri Chaitanya Krishna A. (2023). Color Stability of Pediatric Restorative Material Over Pediatric Drug Formulation. Cureus.

[16] Almutairi M., Moussa I., Alsaeri N., Alqahtani A., Alsulaiman S., Alhajri M. (2022). The effects of different pediatric drugs and brushing on the color stability of esthetic restorative materials used in pediatric dentistry: An in vitro study. Children.

[17] Yıldırım S., Uslu Y.S. (2020). Effects of different pediatric drugs and toothbrushing on color change of restorative materials used in pediatric dentistry. Niger. J. Clin. Pract..

[18] Lindon J.C., Tranter G.E., Holmes J.L. (2000). Encyclopedia of Spectroscopy and Spectrometry.

[19] Khashayar G., Bain P.A., Salari S., Dozic A., Kleverlaan C.J., Feilzer A.J. (2014). Perceptibility and acceptability thresholds for color differences in dentistry. J. Dent..

[20] Korkmaz Y., Ozel E., Attar N., Aksoy G. (2008). The influence of one-step polishing systems on nanocomposites’ surface roughness and microhardness. Oper. Dent..

[21] Dozic A., Kleverlaan C.J., El-Zohainy A., Feilzer A.J., Khashayar G. (2007). Performance of five commercially available tooth color-measuring devices. J. Prosthod..

[22] Yuan K., Sun X., Wang F., Wang H., Chen J.H. (2012). In vitro and in vivo evaluations of three computerized shade matching instruments. Oper. Dent..

[23] Yu B., Ahn J.S., Lee Y.K. (2009). Measurement of translucency of tooth enamel and dentin. Acta Odontol. Scand..

[24] Klotz A.L., Habibi Y., Corcodel N., Rammelsberg P., Hassel A.J., Zenthöfer A. (2022). Laboratory and clinical reliability of two spectrophotometers. J. Esthet. Restor. Dent..

[25] Paravina R.D., Ghinea R., Herrera L.J., Bona A.D., Igiel C., Linninger M., Sakai M., Takahashi H., Tashkandi E., Mar Perez M.D. (2015). Color difference thresholds in dentistry. J. Esthet. Restor. Dent..

[26] Costa C.C., Almeida I.S., Costa Filho L.C. (2006). Erosive effect of an antihistamine-containing syrup on primary enamel and its reduction by fluoride dentifrice. Int. J. Paediatr. Dent..

[27] Maguire A., Baqir W., Nunn J.H. (2007). Are sugars-free medicines more erosive than sugars-containing medicines? An in vitro study of pediatric medicines with prolonged oral clearance used regularly and long-term by children. Int. J. Paediatr. Dent..

[28] Faghihi T., Heidarzadeh Z., Jafari K., Farhoudi I., Hekmatfar S. (2021). An experimental study on the effect of four pediatric drug types on color stability in different tooth-colored restorative materials. Dent. Res. J..

[29] Kathiria H.P., Panda A.K., Virda M., Budakoti V., Dave P.R., Malge R. (2021). Effect of pediatric drugs on color stability of various esthetic restorations in pediatric dentistry. Int. J. Prev. Clin. Dent. Res..

[30] Tüzüner T., Turgut S., Baygin O., Yilmaz N., Tuna E.B., Ozen B. (2017). Effects of Different Pediatric Drugs on the Color Stability of Various Restorative Materials Applicable in Pediatric Dentistry. BioMed Res. Int..

[31] Candan M., Ünal M. (2021). The effect of various asthma medications on surface roughness of pediatric dental restorative materials: An atomic force microscopy and scanning electron microscopy study. Microsc. Res. Tech..

[32] Erdemir U., Yildiz E., Eren M.M., Ozel S. (2013). Surface hardness evaluation of different composite resin materials: Influence of sports and energy drinks immersion after a short-term period. J. Appl. Oral Sci..

[33] Bagheri R., Burrow M.F., Tyas M. (2005). Influence of foodsimulating solutions and surface finish on susceptibility to staining of aesthetic restorative materials. J. Dent..

[34] Tunc E.S., Bayrak S., Guler A.U., Tuloglu N. (2009). The effects of children’s drinks on the color stability of various restorative materials. J. Clin. Pediatr. Dent..

[35] Villalta P., Lu H., Okte Z., Garcia-Godoy F., Powers J.M. (2006). Effects of staining and bleaching on color change of dental composite resins. J. Prosthet. Dent..

[36] Poggio C., Ceci M., Beltrami R., Mirando M., Wassim J., Colombo M. (2016). Color stability of esthetic restorative materials: A spectrophotometric analysis. Acta Biomater. Odontol. Scand..

[37] Alacote-Mauricio B., Gihuaña-Aguilar C., Castro-Ramirez L., Cervantes-Ganoza L., Ladera-Castañeda M., Dapello-Zevallos G., Cayo-Rojas C. (2023). Color Stability in a Giomer, a Conventional Glass Ionomer and a Resin-Modified Glass Ionomer Exposed to Different Pigment Beverages: An in vitro Comparative Study. J. Int. Oral Health.

[38] Ceci M., Viola M., Rattalino D., Beltrami R., Colombo M., Poggio C. (2017). Discoloration of different esthetic restorative materials: A spectrophotometric evaluation. Eur. J. Dent..

[39] Bezgin T., Özer L., Tulga Ö.F., Özkan P. (2015). Effect of toothbrushing on color changes of esthetic restorative materials. J. Esthet. Restor. Dent..

[40] Mohan M., Shey Z., Vaidyanathan J., Vaidyanathan T.K., Munisamy S., Janal M. (2008). Color changes of restorative materials exposed in vitro to cola beverage. Pediatr. Dent..

[41] Hotwani K., Thosar N., Baliga S. (2014). Comparative in vitro assessment of color stability of hybrid esthetic restorative materials against various children’s beverages. J. Conserv. Dent..

[42] Valera B., Bhatt R., Patel M., Patel C., Makwani D., Goyal S. (2022). Effect of Different Pediatric Medications on Various Tooth Colored Restorative Materials Used in Pediatric Dentistry: A Comparative Study. Int. J. Health Sci..

